# Effects of the Artificial Sweetener Neotame on the Gut Microbiome and Fecal Metabolites in Mice

**DOI:** 10.3390/molecules23020367

**Published:** 2018-02-09

**Authors:** Liang Chi, Xiaoming Bian, Bei Gao, Pengcheng Tu, Yunjia Lai, Hongyu Ru, Kun Lu

**Affiliations:** 1 Department of Environmental Sciences and Engineering, University of North Carolina at Chapel Hill, Chapel Hill, NC 27599, USA; liang16@live.unc.edu (L.C.); ptu@live.unc.edu (P.T.); lai7@live.unc.edu (Y.L.); 2 Department of Environmental Health Science, University of Georgia, Athens, GA 30602, USA; bxmroly@uga.edu (X.B.); bgao@uga.edu (B.G.); 3 Department of Population Health and Pathobiology, North Carolina State University, Raleigh, NC 27607, USA; hru@ncsu.edu

**Keywords:** neotame, gut microbiome, metabolomics, artificial sweeteners

## Abstract

Although artificial sweeteners are widely used in food industry, their effects on human health remain a controversy. It is known that the gut microbiota plays a key role in human metabolism and recent studies indicated that some artificial sweeteners such as saccharin could perturb gut microbiome and further affect host health, such as inducing glucose intolerance. Neotame is a relatively new low-caloric and high-intensity artificial sweetener, approved by FDA in 2002. However, the specific effects of neotame on gut bacteria are still unknown. In this study, we combined high-throughput sequencing and gas chromatography–mass spectrometry (GC-MS) metabolomics to investigate the effects of neotame on the gut microbiome and fecal metabolite profiles of CD-1 mice. We found that a four-week neotame consumption reduced the alpha-diversity and altered the beta-diversity of the gut microbiome. Firmicutes was largely decreased while Bacteroidetes was significantly increased. The Phylogenetic Investigation of Communities by Reconstruction of Unobserved States (PICRUSt) analysis also indicated that the control mice and neotame-treated mice have different metabolic patterns and some key genes such as butyrate synthetic genes were decreased. Moreover, neotame consumption also changed the fecal metabolite profiles. Dramatically, the concentrations of multiple fatty acids, lipids as well as cholesterol in the feces of neotame-treated mice were consistently higher than controls. Other metabolites, such as malic acid and glyceric acid, however, were largely decreased. In conclusion, our study first explored the specific effects of neotame on mouse gut microbiota and the results may improve our understanding of the interaction between gut microbiome and neotame and how this interaction could influence the normal metabolism of host bodies.

## 1. Introduction

Artificial sweeteners are important sugar substitutes which are widely used in food and drinks to enhance flavor while avoiding extra energy intake. Some studies indicated artificial sweeteners play a positive role in weight loss, suggesting that it can be employed as a potential dietary tool to assist in weight-loss plan adherence [1,2,3,4]. However, adverse health effects of artificial sweeteners, such as inducing glucose intolerance and causing metabolic syndrome, have been found in recent studies, which indicate that artificial sweeteners have an active metabolic role in the human body and could perturb human metabolism [5,6,7,8]. An epidemiologic study also spotted a positive association between artificial sweetener intake and body weight gain in children [6].

It is known that gut microbiota plays a key regulating role in host metabolism, which is deeply involved in food digestion, energy supplement, and immune system development [9,10]. Environmental factors-induced dysbiosis of gut microbiome is associated with many human diseases, especially obesity, inflammatory bowel disease (IBD) and type 2 diabetes [11,12,13]. In recent years, the influence of artificial sweeteners on gut microbiome have raised concerns as it has been found that many types of artificial sweeteners could perturb the composition of gut bacteria and then affect host health. For example, saccharin could disturb the normal gut microbiota and cause glucose intolerance in rat and human [5]. Our recent study also showed that saccharin could modulate mouse gut microbiota as well as its metabolic functions and induce liver inflammation in mice [14]. Likewise, acesulfame-K (Ace-K) consumption could alter the profile of mouse gut bacteria that is associated with the increase of body weight gain [15].

Neotame (*N*-[*N*-(3,3-dimethylbutyl)-l-α aspartyl]-l-phenylalanine 1-methyl ester) is one of five FDA-approved artificial sweeteners that are 7000–13,000 times sweeter than sugar [16]. In human bodies, neotame can be metabolized by esterase into de-esterified neotame and methanol, and eliminated in the urine and feces within 72 h [16,17]. It has been demonstrated that neotame is well tolerated in many species including Sprague-Dawley CD rats, CD-1 mice, beagle dogs and New Zealand rabbits; similar to other approved artificial sweeteners, neotame is considered as a safe additive to human diets [16]. However, neotame safety studies found that long-term neotame consumption is associated with low body weight and low body weight gain, although this has long been considered the result of low food consumption [18]. In summary, the potential adverse effects of neotame on the gut microbiome, which serves as a key regulating factor to host metabolism, remains unclear and should be addressed.

In this study, we applied 16S rRNA sequencing and GC-MS metabolomics to investigated the effects of neotame on the gut microbiome and the fecal metabolome of male CD-1 mice. This study may provide important findings towards a better understanding of the impact of artificial sweetener consumption on human health.

## 2. Results

### 2.1. Neotame Consumption Altered Diversities and Component Profiles of the Gut Microbiome in CD-1 Mice

**Figure 1:**
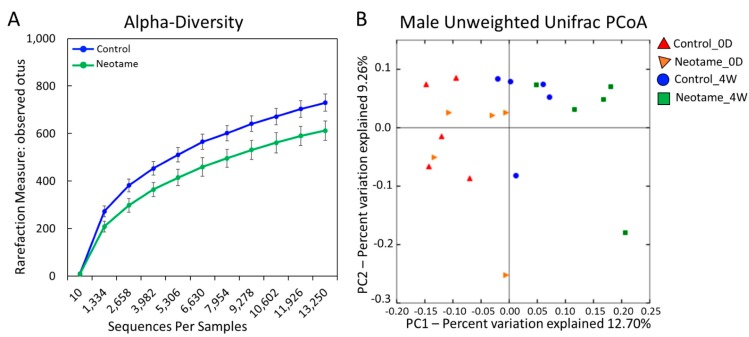
(**A**) The alpha-diversity of gut microbiome in neotame-treated mice was significantly lower than controls; (**B**) The PCoA analysis (beta-diversity) indicated a difference of gut microbiome communities in control and treated mice after four-week neotame consumption.

**Figure 2:**
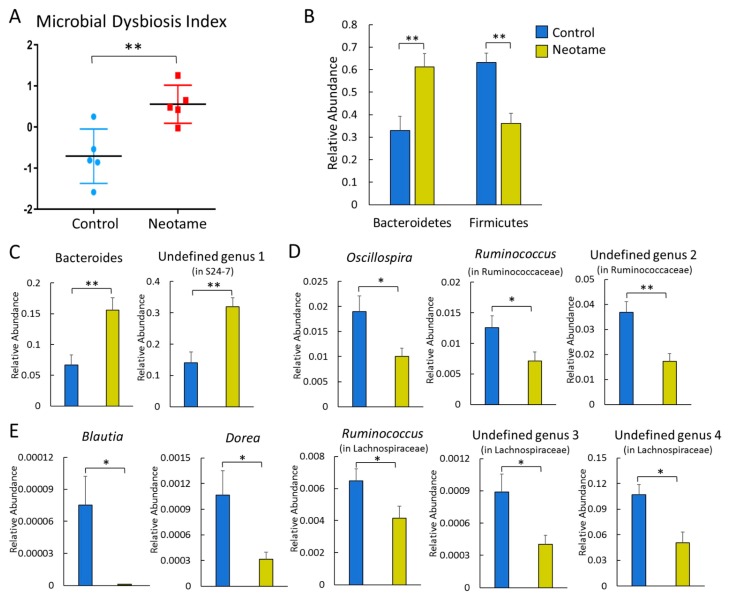
(**A**) Neotame-treated gut microbiome (*n* = 5) has a significant higher MD-index than controls (*n* = 5); (**B**) Phylum Bacteroidetes has been enriched in neotame-treated mice, while Firmicutes has been reduced; (**C**) Two main altered genera in phylum Bacteroidetes; (**D**) Significantly decreased three genera in family Ruminococcaceae; (**E**) Significantly decreased five genera in family Lachnospiraceae. (* *p* < 0.05; ** *p* < 0.01).

We first investigated whether a four-week neotame consumption would affect the gut microbiome of CD-1 mouse. No significant difference of alpha-diversity was observed between two groups before neotame consumption. After the four-week experiment, alpha-diversity of gut microbiome in neotame-consuming group was much lower than the control group, as shown in Figure 1A. PCoA analysis showed a separation of gut bacteria between control and neotame-consuming animals after the four-week treatment, compared to their clustering distribution before neotame consumption (Figure 1B). The results suggest that neotame consumption significantly altered both the alpha- and beta-diversities of gut bacteria of mice. Dysbiosis analysis found that neotame-treated mice had a significantly higher microbial dysbiosis index (MD-index) than controls (Figure 2A). Specifically, phylum Bacteroidetes was largely enriched, while Firmicutes was significantly decreased in neotame-treated animals (Figure 2B). Before neotame treatment, no such significant taxonomy difference was observed on the phylum level. On genus levels, we found that *Bacteroides* and an undefined genus in family S24-7 mainly contributed to the increase of phylum Bacteroidetes, as shown in Figure 2C. Over 12 genera have been significantly altered in Firmicutes (Table S1). Notably, multiple components of family Lachnospiraceae and family Ruminococcaceae in neotame-treated animals were significantly lower than controls, such as *Blautia*, *Dorea*, *Oscillospira* and *Ruminococcus* (Figure 2D,E). More altered genus can be found in Table S1 (Supplementary Materials). Taken together, the results suggested that the four-week neotame consumption perturbed the diversities as well as the community compositions of gut microbiome in male CD-1 mice.

### 2.2. Neotame Consumption Altered the Metabolic Pathway Pattern of Gut Microbiome

**Figure 3:**
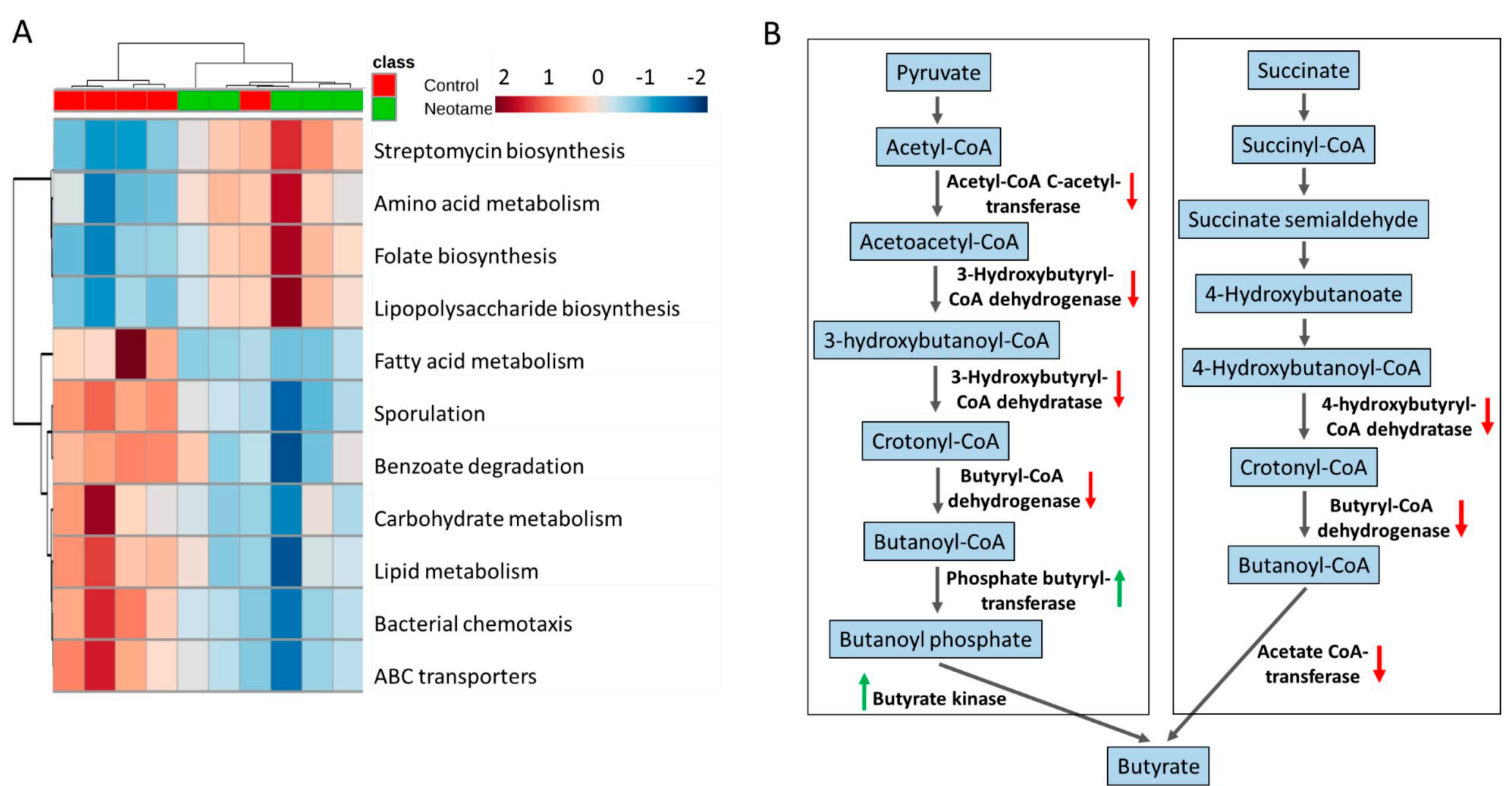
(**A**) The pattern of some key metabolic pathways in control (*n* = 5) and neotame-treated mice (*n* = 5) were different; (**B**) Multiple genes in two butyrate biosynthetic pathways have been decreased in neotame-treated mice.

The perturbation of gut bacteria composition generally alters the functional gene profile. Therefore, we further investigated whether neotame consumption altered functional pathways in gut microbiome. As shown in Figure 3A, neotame-treated gut microbiota shows a different pattern of metabolic pathways compared to controls. Specifically, in the neotame-treated microbiome, amino acid metabolism, LPS biosynthesis, antibiotics biosynthesis and folate biosynthesis pathways were enriched. However, for the abundances of pathways, such as fatty acid metabolism, carbohydrate metabolism, lipid metabolism and ABC transporters, they are generally lower than in controls. Besides, we found multiple genes in two classical butyrate fermentation pathways have been significantly reduced (Figure 3B). Three genes, which encode 4-hydroxybutyryl-CoA dehydratase, butyryl-CoA dehydrogenase and acetate CoA-transferase, respectively while participating in the succinate fermentation to butyrate, were significantly decreased. For the other pathway of butyrate fermented from pyruvate, although the genes of phosphate butyryltransferase and butyrate kinase were increased, four upstream genes were significantly reduced, including acetyl-CoA C-acetyltransferase, 3-hydroxybutyryl-CoA dehydrogenase, 3-hydroxybutyryl-CoA dehydratase and butyryl-CoA dehydratase and butyryl-CoA dehydrogenase (Figure 3B).

### 2.3. Neotame Altered Metabolite Profiles in Fecal Samples of Mice

**Figure 4:**
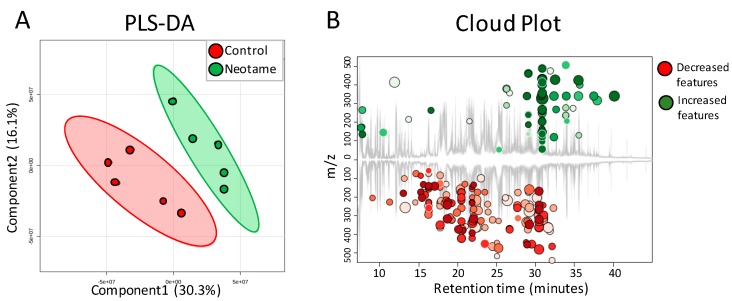
(**A**) PLS-DA analysis shows that the fecal metabolite profiles are different between control (*n* = 5) and neotame-treated animals (*n* = 5); (**B**) Cloud Plot gives the ion features that have significantly different (*p* ≤ 0.05, fold change ≥1.5) abundance between control and neotame-treated animals.

**Figure 5:**
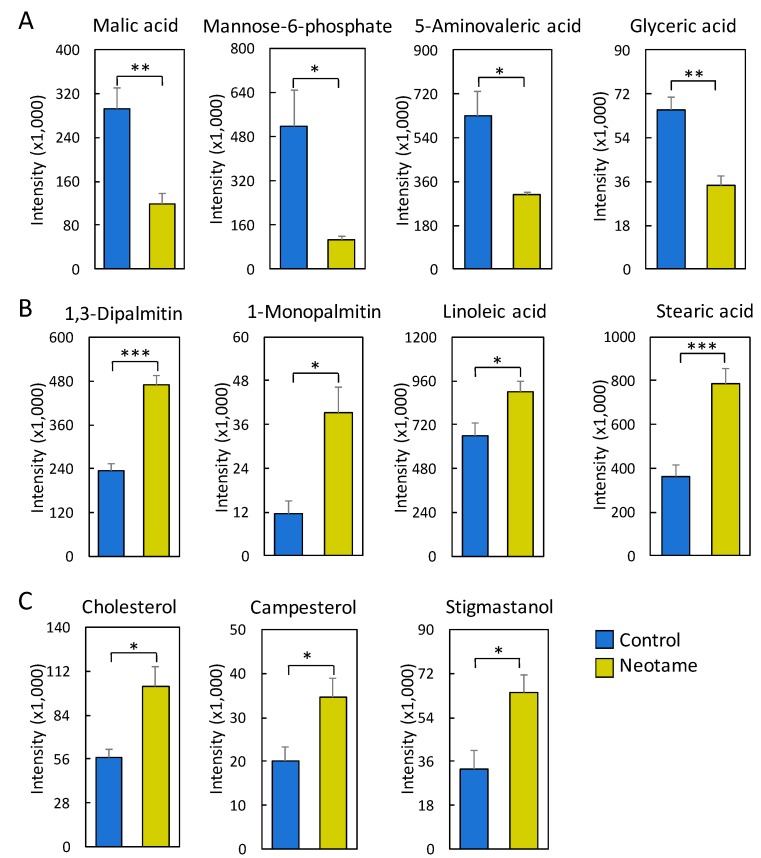
(**A**) Malic acid, mannose-6-phosphate, 5-aminovaleric acid and glyceric acid are significantly reduced in the fecal samples of neotame-treated mice; (**B**) Multiple fatty acids and lipids are significantly increased in the fecal samples of neotame-treated mice; (**C**) Cholesterol, campesterol and stigmastanol are significantly increased in the fecal samples of neotame-treated mice. (* *p* < 0.05; ** *p* < 0.01; *** *p* < 0.001).

We further investigated whether neotame consumption could perturb the fecal metabolome of the mouse. As we predicted, along with the perturbed gut microbiota, fecal metabolite profiles were also largely altered, as shown in Figure 4A,B. Most of the altered metabolites were decreased in neotame-treated mice, such as malic acid, mannose-6-phosphate, 5-aminovaleric acid and glyceric acid (Figure 5A). Interestingly, we found most of the identified lipids and fatty acids were significantly decreased in treated mice, including 1,3-dipalmitate, 1-monopalmitin, linoleic acid and stearic acid (Figure 5B). Moreover, the concentrations of cholesterol, campesterol and stigmastanol were also increased in the fecal samples of neotame consumption, as shown in Figure 5C. More altered metabolites can be found in Table S2.

## 3. Discussion

Artificial sweeteners generally cannot be utilized by human bodies. For a long time, they have been considered as safe food additives with a negligible influence on the normal metabolism of human. However, recent studies indicated that some of artificial sweeteners could perturb gut microbiota in mammals and further affect the host health, such as inducing glucose intolerance [5,14]. The results of our current study clearly demonstrated that a four-week neotame consumption disturbed the mouse gut microbiome. Notably, we found Bacteroidetes was largely enriched, mainly due to the increases of genus *Bacteroides* and a genus in family *S24-7* (Figure 2B,C). According to previous studies, other artificial sweeteners such as saccharin and Ace-k also could induce a promoted growth of Bacteroides [5,15]. As one of the most abundant bacteria in mammal gut, *Bacteroides* plays important roles in glycan digestion and polysaccharide fermentation [19,20]. *S24-7*, also called *Candidatus Homeothermaceae*, is a substantial component of mouse and human gut microbiota, and metagenomics sequencing indicates the genome of *S24-7* contains polysaccharide utilizing genes and multiple vitamin synthetic genes [21], which is similar for *Bacteroides*. The upregulation of some pathways like folate synthesis and LPS biosynthesis should mainly come from the improvements of *Bacteroides* and *S24-7* (Figure 3A).

On the other hand, however, neotame consumption extensively induced the decline of various components in Firmicutes, which corresponded to the reduced alpha-diversity (Figure 1 and Figure 2). Notably, multiple genera in family Lachnospiraceae and Ruminococcaceae were significantly decreased (Figure 2D,E). As the important components of Firmicutes, Lachnospiraceae and Ruminococcaceae have been considered as two of major plant degraders and short-chain fatty acid (SCFA) producers, with critical roles in host nutrition supplement and energy homeostasis [22,23]. This is in alignment with the downregulation of carbohydrate metabolism pathways and multiple butyrate synthetic genes in the gut microbiome of neotame-consuming mice (Figure 3). To conclude, the extensive decline of Lachnospiraceae and Ruminococcaceae as well as other components of Firmicutes may reduce the energy-harvesting capacity of gut microbiome and therefore, influence host energy homeostasis, which is correlated to the observed low body weight gain in previous chronic neotame safety studies [18,24].

The ratio of Firmicutes and Bacteroidetes (F/B) has been widely studied regarding their associations with obesity. However, previous studies have very inconsistent conclusions. For example, some studies found high F/B ratio in the microbiome of obese subjects [11,25], while other studies reported a reverse trend or even a negligible difference of F/B ratio between obese and lean subjects [26,27,28]. Nevertheless, it is clear that components in Firmicuties and Bacteroidetes may share some functions. For example, *Bacteroides* (a Bacteroidetes) is known as an important plant polysaccharides degrader, as is *Ruminococcus* (a Firmicuties) [29,30]. For this study, the alterations in functional pathways and fecal metabolite profiles may reflect the overall functional effects of neotame on gut microbiome, as the functional damage caused by extensive decrease of Firmicuties could be partially complemented by the increase of Bacteroidetes.

Fecal metabolome data further confirmed the observed effects of neotame consumption on gut microbiota. As shown in Figure 4, the concentrations of hundreds of metabolites have undergone a significant decrease in the feces of treated mice, including malic acid, mannose-6-phosphate and glyceric acid (Figure 4 and Figure 5A). The decreased concentrations of these metabolites may be caused by the decline of Firmicutes (Figure 2B). Conversely, we found that the concentrations of most of the identified lipids and fatty acids in feces of neotame-consuming mice were higher than controls, including linoleic acid, stearic acid, 1-monopalmitin and 1,3-dipalmitate (Figure 5B). It seems that the absorption of those lipids and fatty acids in neotame-treated mice are lower than controls, which may partially explain the observed neotame-induced low body weight gain in previous neotame safety studies [18]. It is established that gut microbiota plays an important role in host lipid and fatty acid absorption and metabolism [31,32]. A recent research has demonstrated that Firmicutes could promote the fatty acid absorption and increase epithelial lipid droplet in Zebrafish [33]. In this study, neotame consumption largely reduced the abundance of Firmicutes, which may result in a lowered absorbing efficiency of fatty acids and lipids and increased levels of them in feces. However, previous studies also proposed other potential explanations. First, it has been found that SCFAs could inhibit gastric motility by increasing peptide YY levels [34,35,36]. The decline in gastric motility allows for more intestinal epithelial contact time and therefore increase energy absorbing efficiency [37]. For our current study, the large decrease of Lachnospiraceae and Ruminococcaceae and the reduced butyrate synthetic genes upon neotame-induced perturbation of gut microbiome may indicate a decline in SCFA production, thereby reducing lipid and fatty acid absorption. Moreover, previous study indicated that SCFAs could promote the L-cell differentiation and increase the L-cell number, which increased the glucagon-like peptide 1 (GLP-1) release [38]. The decrease of SCFAs production might influence the GLP-1 release, which can deeply affect the lipid metabolism [39]. Besides, it is known that gut microbiota also influences the bile acids metabolism, which is essential in lipid and cholesterol metabolism [40,41,42]. In this regard, the perturbation of gut bacteria may lead to an altered bile acid metabolism and further influence the absorption of lipids and fatty acids. The increased levels of cholesterol, campesterol and stigmastanol may share similar mechanisms with the alterations of fatty acids and lipids; however, our current data cannot demonstrate such a causation. More work needs to be done in future studies to reveal the mechanism of how neotame affects the fecal profiles of fatty acids and lipids.

Metabolic effects of the artificial sweetener neotame are still poorly understood. This study for the first time investigates the effects of neotame consumption on mouse gut microbiome. The results indicate negative effects on gut microbiota in mice that the use of neotame can induce perturbation in gut bacteria, including bacterial community compositions, functional genes, and the metabolome. The results yielded in this study may provide insights towards an improved mechanistic understanding of the interaction of neotame, the gut microbiome and host metabolism, and may be useful to resolving the much controversial health impacts of artificial sweeteners.

The current research has several limitations. First, the sample size in this study is relative small, that future studies need to further validate the effects of neotame on gut microbiome using a larger number of animals or a human cohort. Second, our results are based on 16S sequencing, PICRUSt analysis, and non-target metabolomics. However, shotgun metagenomics sequencing and target analysis especially for metabolites like SCFAs might have yielded better results. Moreover, the current study is a four-week neotame exposure, while human exposure is frequently long term and at lower concentrations. Future studies are necessary to explore effects of long-term exposure in human.

## 4. Materials and Methods

### 4.1. Animals and Neotame Treatment

Ten male CD-1 mice around 7 weeks old were purchased from Charles River Laboratories and allowed to acclimate for 1 week prior to use. All the mice were housed in the University of Georgia animal facility with 22 °C, 40–70% humidity and a 12:12 h light:dark cycle. A standard pelleted rodent diet and tap water ad libitum were supplied. The mice were randomly assigned to the control and neotame treated groups (5 mice for control group; 5 mice for neotame treated group). Clean water (control group) or neotame-containing water (neotame treated group) were administered to the mice by gavage for 4 weeks. The dose (0.75 mg/kg body weight/day) we used was equivalent to or much lower than those adopted in former studies [24,43]. The dose (0.75 mg/kg body weight/day) was 2.5× human allowed daily intake (ADI) (0.3 mg/kg body weight/day) for neotame set by the U.S. FDA. The use of 2.5× human ADI allowed us to compare the effects of neotame on the gut microbiome with another artificial sweetener study which also used 2.5× human ADI for animal exposure [15]. If we use 12.3 as the exchange factor between human and mouse [44], the equivalent dose for human is around 0.06 mg/kg body weight/day. Mice were weighted before and after neotame treatment. Mouse body weight did not have significant difference between control and treatment group. No difference of eating behavior and other behaviors were observed between two groups. Fecal samples were collected before neotame treatment and after four-week treatment and were frozen in liquid nitrogen and stored at −80 °C. At the end of the experiment, mice were euthanized with CO_2_ in an appropriate chamber by trained personnel. The University of Georgia Institutional Animal Care and Use Committee reviewed and approved all of operations and processes adopted in this experiment (protocol ID: A2014 10-014-Y2-A1).

### 4.2. 16S rRNA Gene Sequencing

The sequencing library was built by the method as previously described [15]. Briefly, fecal DNA (~1 ng) was isolated by a PowerSoil DNA Isolation Kit (Mo Bio Laboratories). The V4 region of the 16S rRNA gene was amplified by the universal primers 515F (5′-GTGCCAGCMGCCGCGGTAA) and 806R (5′-GGACTACHVGGGTWTCTAAT). Each individual sample was barcoded by a unique sequence, and then pooled together and sequenced by Illumina MiSeq at the Georgia Genomics Facility (PE250, v2 kit). The raw sequencing files were set-paired, trimmed (with 0.01 error probability limit) and merged by Geneious 8.0.5 (Biomatters, Auckland, New Zealand). Quantitative insights into microbial ecology (QIIME, version 1.9.1) was used for further analysis and the operational taxonomic units (OTUs) was obtained by UCLUST with 97% sequence similarity [45]. 

### 4.3. Functional Gene Enrichment Analysis

PICRUSt (Galaxy Version 1.0.0, http://huttenhower.sph.harvard.edu/galaxy/) was applied to analyze the enrichment of functional pathways and genes in the gut microbiome [46]. PICRUSt uses the marker genes in 16S sequencing data to match the reference genome database to estimate the gene profiles of bacterial communities [47,48,49]. PICRUSt result was input into the Statistical Analysis of Metagenomic Profiles (STAMP) (version 2.1.3) for statistical analysis [50].

### 4.4. Metabolomics Analysis

Fecal metabolites were extracted using the method as described previously [51]. Briefly, 1 mL of methanol/chloroform/water solution (2:2:1) added to 20 mg of feces and vortexed for 1 h. Then, centrifuging at 3200× *g* for 15 min and then transferred the upper and lower phases to vials. Solvent was dried in a SpeedVac, and metabolites were derivatized using *N*,*O*-Bis(trimethylsilyl)trifluoroacetamide (BSTFA). Metabolomics profiling was harvested by an Agilent 6890 GC coupled to a 5973 MSD system. One microliter of sample extract was injected. The carrier gas is Helium (99.9999%) and the flow rate was set as 0.8 mL min^−1^. A 30 m × 0.25 mm diameter DB-5ms column (Agilent, Santa Clara, CA, USA) was used in this study. The oven temperature was started at 60 °C holding for 2 min, and then raised to 320 °C at 8 °C min^−1^ and held for 10.5 min. The equilibration time is 0.5 min. Metabolite features with mass range from *m*/*z* 50 to 600 were captured. The data files were processed with XCMS Online (https://xcmsonline.scripps.edu/) to pick and align peaks, and calculate peak intensities. The “GC/Single Quad (centWave)” was selected as the parameter set. The *m*/*z* tolerance was 100 ppm. Unpaired parametric Welch’s *t*-test was applied to assess the differences of features between two groups and the *p*-value threshold was set to 0.05. The default values of other parameters in the “GC/Single Quad (centWave)” were adopted. Features with significant changes (*p* < 0.05, intensity > 1000) were selected and identified by matching with the National Institute of Standards and Technology (NIST) Standard Reference Database.

### 4.5. Statistical Analysis

Microbial dysbiosis index was calculated by QIIME based on the approach described in a previous study [52]. The metastats command in mothur software was used to calculate the difference in the gut microbiota taxonomy between control and treated groups [53,54]. Besides, the differences in the fecal metabolomes of the control and neotame-treated groups was assessed by partial least squares discriminant analysis (PLS-DA), and the differences in functional genes and the fecal metabolites was assessed by the two-tailed Welch’s *t*-test (*p* < 0.05). 

## Supplementary Materials

Supplementary materials are available online.

## References

[1] Fitch C., Keim K.S. (2012). Position of the Academy of Nutrition and Dietetics: Use of nutritive and nonnutritive sweeteners. J. Acad. Nutr. Diet..

[2] Morris D.H., Cuneo P., Stuart M.J., Mance M.J., Bell K.J., Puleo E., Ahmadi S., Ward A., Rippe J.M. (1993). High-intensity sweetener, energy and nutrient intakes of overweight women and men participating in a weight-loss program. Nutr. Res..

[3] Bellisle F., Drewnowski A. (2007). Intense sweeteners, energy intake and the control of body weight. Eur. J. Clin. Nutr..

[4] Husøy T., Mangschou B., Fotland T., Kolset S., Jakobsen H.N., Tømmerberg I., Bergsten C., Alexander J., Andersen L.F. (2008). Reducing added sugar intake in Norway by replacing sugar sweetened beverages with beverages containing intense sweeteners—A risk benefit assessment. Food Chem. Toxicol..

[5] Suez J., Korem T., Zeevi D., Zilberman-Schapira G., Thaiss C.A., Maza O., Israeli D., Zmora N., Gilad S., Weinberger A. (2014). Artificial sweeteners induce glucose intolerance by altering the gut microbiota. Nature.

[6] Brown R.J., De Banate M.A., Rother K.I. (2010). Artificial sweeteners: A systematic review of metabolic effects in youth. Int. J. Pediatr. Obes..

[7] Dhingra R., Sullivan L., Jacques P.F., Wang T.J., Fox C.S., Meigs J.B., D’Agostino R.B., Gaziano J.M., Vasan R.S. (2007). Soft drink consumption and risk of developing cardiometabolic risk factors and the metabolic syndrome in middle-aged adults in the community. Circulation.

[8] Fowler S.P., Williams K., Resendez R.G., Hunt K.J., Hazuda H.P., Stern M.P. (2008). Fueling the obesity epidemic? Artificially sweetened beverage use and long-term weight gain. Obesity.

[9] Kau A.L., Ahern P.P., Griffin N.W., Goodman A.L., Gordon J.I. (2011). Human nutrition, the gut microbiome and the immune system. Nature.

[10] Nicholson J.K., Holmes E., Kinross J., Burcelin R., Gibson G., Jia W., Pettersson S. (2012). Host-gut microbiota metabolic interactions. Science.

[11] Turnbaugh P.J., Ley R.E., Mahowald M.A., Magrini V., Mardis E.R., Gordon J.I. (2006). An obesity-associated gut microbiome with increased capacity for energy harvest. Nature.

[12] Qin J., Li Y., Cai Z., Li S., Zhu J., Zhang F., Liang S., Zhang W., Guan Y., Shen D. (2012). A metagenome-wide association study of gut microbiota in type 2 diabetes. Nature.

[13] Kostic A.D., Xavier R.J., Gevers D. (2014). The microbiome in inflammatory bowel disease: Current status and the future ahead. Gastroenterology.

[14] Bian X., Tu P., Chi L., Gao B., Ru H., Lu K. (2017). Saccharin induced liver inflammation in mice by altering the gut microbiota and its metabolic functions. Food Chem. Toxicol..

[15] Bian X., Chi L., Gao B., Tu P., Ru H., Lu K. (2017). The artificial sweetener acesulfame potassium affects the gut microbiome and body weight gain in CD-1 mice. PLoS ONE.

[16] Satyavathi K., Raju P.B., Bupesh K., Kiran T.N.R. (2010). Neotame: High intensity low caloric sweetener. Asian J. Chem..

[17] Whitehouse C.R., Boullata J., McCauley L.A. (2008). The potential toxicity of artificial sweeteners. AAOHN J..

[18] Flamm W.G., Blackburn G.L., Comer C.P., Mayhew D.A., Stargel W.W. (2003). Long-term food consumption and body weight changes in neotame safety studies are consistent with the allometric relationship observed for other sweeteners and during dietary restrictions. Regul. Toxicol. Pharmacol..

[19] Wexler H.M. (2007). Bacteroides: The good, the bad, and the nitty-gritty. Clin. Microbiol. Rev..

[20] Johnson E.L., Heaver S.L., Walters W.A., Ley R.E. (2016). Microbiome and metabolic disease: Revisiting the bacterial phylum Bacteroidetes. J. Mol. Med..

[21] Ormerod K.L., Wood D.L., Lachner N., Gellatly S.L., Daly J.N., Parsons J.D., Dal’Molin C.G., Palfreyman R.W., Nielsen L.K., Cooper M.A. (2016). Genomic characterization of the uncultured Bacteroidales family S24–S7 inhabiting the guts of homeothermic animals. Microbiome.

[22] Meehan C.J., Beiko R.G. (2014). A phylogenomic view of ecological specialization in the *Lachnospiraceae*, a family of digestive tract-associated bacteria. Genome Biol. Evol..

[23] Biddle A., Stewart L., Blanchard J., Leschine S. (2013). Untangling the genetic basis of fibrolytic specialization by Lachnospiraceae and Ruminococcaceae in diverse gut communities. Diversity.

[24] Mayhew D.A., Comer C.P., Stargel W.W. (2003). Food consumption and body weight changes with neotame, a new sweetener with intense taste: Differentiating effects of palatability from toxicity in dietary safety studies. Regul. Toxicol. Pharmacol..

[25] Ley R.E., Turnbaugh P.J., Klein S., Gordon J.I. (2006). Microbial ecology: Human gut microbes associated with obesity. Nature.

[26] Duncan S.H., Lobley G., Holtrop G., Ince J., Johnstone A., Louis P., Flint H. (2008). Human colonic microbiota associated with diet, obesity and weight loss. Int. J. Obes..

[27] Zhang H., DiBaise J.K., Zuccolo A., Kudrna D., Braidotti M., Yu Y., Parameswaran P., Crowell M.D., Wing R., Rittmann B.E. (2009). Human gut microbiota in obesity and after gastric bypass. Proc. Natl. Acad. Sci. USA.

[28] Jumpertz R., Le D.S., Turnbaugh P.J., Trinidad C., Bogardus C., Gordon J.I., Krakoff J. (2011). Energy-balance studies reveal associations between gut microbes, caloric load, and nutrient absorption in humans. Am. J. Clin. Nutr..

[29] Schwiertz A., Taras D., Schäfer K., Beijer S., Bos N.A., Donus C., Hardt P.D. (2010). Microbiota and SCFA in lean and overweight healthy subjects. Obesity.

[30] Robert C., Bernalier-Donadille A. (2003). The cellulolytic microflora of the human colon: Evidence of microcrystalline cellulose-degrading bacteria in methane-excreting subjects. FEMS Microbiol. Ecol..

[31] Velagapudi V.R., Hezaveh R., Reigstad C.S., Gopalacharyulu P., Yetukuri L., Islam S., Felin J., Perkins R., Borén J., Orešič M. (2010). The gut microbiota modulates host energy and lipid metabolism in mice. J. Lipid Res..

[32] Caesar R., Fåk F., Bäckhed F. (2010). Effects of gut microbiota on obesity and atherosclerosis via modulation of inflammation and lipid metabolism. J. Intern. Med..

[33] Semova I., Carten J.D., Stombaugh J., Mackey L.C., Knight R., Farber S.A., Rawls J.F. (2012). Microbiota regulate intestinal absorption and metabolism of fatty acids in the zebrafish. Cell Host Microbe.

[34] Cuche G., Cuber J., Malbert C.-H. (2000). Ileal short-chain fatty acids inhibit gastric motility by a humoral pathway. Am. J. Physiol. Gastrointest. Liver Physiol..

[35] Samuel B.S., Shaito A., Motoike T., Rey F.E., Backhed F., Manchester J.K., Hammer R.E., Williams S.C., Crowley J., Yanagisawa M. (2008). Effects of the gut microbiota on host adiposity are modulated by the short-chain fatty-acid binding G protein-coupled receptor, Gpr41. Proc. Natl. Acad. Sci. USA.

[36] Krajmalnik-Brown R., Ilhan Z.-E., Kang D.-W., DiBaise J.K. (2012). Effects of gut microbes on nutrient absorption and energy regulation. Nutr. Clin. Pract..

[37] Taylor I.L. (1993). Role of peptide YY in the endocrine control of digestion. J. Dairy Sci..

[38] Petersen N., Reimann F., Bartfeld S., Farin H.F., Ringnalda F.C., Vries R.G., van den Brink S., Clevers H., Gribble F.M., de Koning E.J. (2014). Generation of L cells in mouse and human small intestine organoids. Diabetes.

[39] Farr S., Taher J., Adeli K. (2014). Glucagon-like peptide-1 as a key regulator of lipid and lipoprotein metabolism in fasting and postprandial states. Cardiovasc. Haematol. Disord. Drug Targets.

[40] Swann J.R., Want E.J., Geier F.M., Spagou K., Wilson I.D., Sidaway J.E., Nicholson J.K., Holmes E. (2011). Systemic gut microbial modulation of bile acid metabolism in host tissue compartments. Proc. Natl. Acad. Sci. USA.

[41] Ridlon J.M., Kang D.J., Hylemon P.B., Bajaj J.S. (2014). Bile acids and the gut microbiome. Curr. Opin. Gastroenterol..

[42] Trauner M., Claudel T., Fickert P., Moustafa T., Wagner M. (2010). Bile acids as regulators of hepatic lipid and glucose metabolism. Dig. Dis..

[43] Zhu L., Wang G., Dong B., Peng C., Tian Y., Gong L. (2016). Effects of sweetener neotame on diet preference, performance and hematological and biochemical parameters of weaned piglets. Anim. Feed Sci. Technol..

[44] Nair A.B., Jacob S. (2016). A simple practice guide for dose conversion between animals and human. J. Basic Clin. Pharm..

[45] Caporaso J.G., Kuczynski J., Stombaugh J., Bittinger K., Bushman F.D., Costello E.K., Fierer N., Pena A.G., Goodrich J.K., Gordon J.I. (2010). QIIME allows analysis of high-throughput community sequencing data. Nat. Methods.

[46] Langille M.G., Zaneveld J., Caporaso J.G., McDonald D., Knights D., Reyes J.A., Clemente J.C., Burkepile D.E., Thurber R.L.V., Knight R. (2013). Predictive functional profiling of microbial communities using 16S rRNA marker gene sequences. Nat. Biotechnol..

[47] Garcia-Mazcorro J.F., Castillo-Carranza S.A., Guard B., Gomez-Vazquez J.P., Dowd S.E., Brigthsmith D.J. (2017). Comprehensive molecular characterization of bacterial communities in feces of pet birds using 16S marker sequencing. Microb. Ecol..

[48] Bunyavanich S., Shen N., Grishin A., Wood R., Burks W., Dawson P., Jones S.M., Leung D.Y., Sampson H., Sicherer S. (2016). Early-life gut microbiome composition and milk allergy resolution. J. Allergy Clin. Immunol..

[49] Sheflin A.M., Borresen E.C., Kirkwood J.S., Boot C.M., Whitney A.K., Lu S., Brown R.J., Broeckling C.D., Ryan E.P., Weir T.L. (2016). Dietary supplementation with rice bran or navy bean alters gut bacterial metabolism in colorectal cancer survivors. Mol. Nutr. Food Res..

[50] Parks D.H., Tyson G.W., Hugenholtz P., Beiko R.G. (2014). STAMP: Statistical analysis of taxonomic and functional profiles. Bioinformatics.

[51] Lu K., Ryan P.A., Schlieper K.A., Graffam M.E., Levine S., Wishnok J.S., Swenberg J.A., Tannenbaum S.R., Fox J.G. (2014). Arsenic exposure perturbs the gut microbiome and its metabolic profile in mice: An integrated metagenomics and metabolomics analysis. Environ. Health Perspect..

[52] Gevers D., Kugathasan S., Denson L.A., Vázquez-Baeza Y., Van Treuren W., Ren B., Schwager E., Knights D., Song S.J., Yassour M. (2014). The treatment-naive microbiome in new-onset Crohn’s disease. Cell Host Microbe.

[53] Schloss P.D., Westcott S.L., Ryabin T., Hall J.R., Hartmann M., Hollister E.B., Lesniewski R.A., Oakley B.B., Parks D.H., Robinson C.J. (2009). Introducing mothur: Open-source, platform-independent, community-supported software for describing and comparing microbial communities. Appl. Environ. Microbiol..

[54] White J.R., Nagarajan N., Pop M. (2009). Statistical methods for detecting differentially abundant features in clinical metagenomic samples. PLoS Comput. Biol..

