# Supplementary material for: Effects of the Artificial Sweetener Neotame on the Gut Microbiome and Fecal Metabolites in Mice
Source: Molecules. 2018 Feb 9;23(2):367. doi: 10.3390/molecules23020367 (PMC6017827; doi:10.3390/molecules23020367)
Supplement: Supplementary file 1 [file molecules-23-00367-s001.pdf]

Supplemental Materials

Effects of the Artificial Sweetener Neotame on the Gut Microbiome and Fecal Metabolites in Mice

Liang Chi[1], Xiaoming Bian[2], Bei Gao[2], Pengcheng Tu[1], Yunjia Lai [1], Hongyu Ru[3], and Kun Lu[1,*]

1. Department of Environmental Sciences and Engineering,

University of North Carolina at Chapel Hill, 27599, United States

2. Department of Environmental Health Science,

University of Georgia, Athens, Georgia 30602, United States

3. Department of Population Health and Pathobiology,

North Carolina State University, Raleigh, NC, 27607

* Corresponding Authors

Email: kunlu@unc.edu

Table S1. Significantly different gut bacteria genera between the control and 4-week neotame consumed mice

Taxa	Control mice		Neotame-treated mice		Fold change	p- value
	Relative abundance	Variance	Relative abundance	Variance		
B1	0.0667	0.00130	0.1562	0.00196	2.343	0.0070
B2	0.1402	0.00590	0.3195	0.00419	2.280	0.0035
B3	0.0246	0.00048	0.0030	0.00000	-8.088	0.0480
B4	0.4130	0.01848	0.1988	0.00382	-2.078	0.0112
B5	0.0024	0.00001	0.0078	0.00001	3.180	0.0176
B6	0.0014	0.00000	0.0007	0.00000	-2.097	0.0307
B7	0.0009	0.00000	0.0004	0.00000	-2.214	0.0263
B8	0.1068	0.00071	0.0504	0.00084	-2.117	0.0115
B9	0.0001	0.00000	0.0000	0.00000	-	0.0196
B10	0.0011	0.00000	0.0003	0.00000	-3.390	0.0269
B11	0.0065	0.00000	0.0042	0.00000	-1.559	0.0474
B12	0.0368	0.00010	0.0173	0.00005	-2.128	0.0055
B13	0.0190	0.00005	0.0101	0.00001	-1.887	0.0276
B14	0.0126	0.00002	0.0071	0.00001	-1.759	0.0470
B15	0.0006	0.00000	0.0001	0.00000	-4.982	0.0027

*The abbreviations of bacteria genus are listed as below (k, bacteria; p, phylum; c, class; o, order; f, family; g, genus):

B1: k__Bacteria;p__Bacteroidetes;c__Bacteroidia;o__Bacteroidales;f__Bacteroidaceae;g__Bacteroides;
B2: k__Bacteria;p__Bacteroidetes;c__Bacteroidia;o__Bacteroidales;f__S24-7;g__Undefined;
B3: k__Bacteria;p__Deferribacteres;c__Deferribacteres;o__Deferribacterales;f__Deferribacteraceae; g__Mucispirillum;
B4: k__Bacteria;p__Firmicutes;c__Clostridia;o__Clostridiales;f__Undefined;g__Undefined;
B5: k__Bacteria;p__Firmicutes;c__Clostridia;o__Clostridiales;f__Clostridiaceae; g__CandidatusArthromitus;
B6: k__Bacteria;p__Firmicutes;c__Clostridia;o__Clostridiales;f__Dehalobacteriaceae; g__Dehalobacterium;
B7: k__Bacteria;p__Firmicutes;c__Clostridia;o__Clostridiales;f__Lachnospiraceae;g_undefined;
B8: k__Bacteria;p__Firmicutes;c__Clostridia;o__Clostridiales;f__Lachnospiraceae;g__Undefined;
B9: k__Bacteria;p__Firmicutes;c__Clostridia;o__Clostridiales;f__Lachnospiraceae;g__Blautia;

B10: k__Bacteria;p__Firmicutes;c__Clostridia;o__Clostridiales;f__Lachnospiraceae;g__Dorea;
B11: k__Bacteria;p__Firmicutes;c__Clostridia;o__Clostridiales;f__Lachnospiraceae;g__Ruminococcus;
B12: k__Bacteria;p__Firmicutes;c__Clostridia;o__Clostridiales;f__Ruminococcaceae;g__Undefined;
B13: k__Bacteria;p__Firmicutes;c__Clostridia;o__Clostridiales;f__Ruminococcaceae;g__Oscillospira;
B14: k__Bacteria;p__Firmicutes;c__Clostridia;o__Clostridiales;f__Ruminococcaceae;g__Ruminococcus;
B15: k__Bacteria;p__Firmicutes;c__Clostridia;o__Clostridiales;f__Mogibacteriaceae;g__Undefined.

Supplemental Material, Table S2. Identified significantly changed metabolites ($p < 0.05$, compared to controls) in fecal samples of neotame consumed mice.

Metabolites	Intensity (Control)	Intensity (Neotame)	Fold_change	p value
α-Tocopherol	17918.40	52139.20	2.91	0.0296
1,3-Dipalmitin	234277.16	470764.74	2.01	0.0001
7H-purine	128324.10	65536.59	-1.96	0.0290
Linoleic acid	659558.59	897080.28	1.36	0.0447
9H-Purine	291689.20	105043.81	-2.78	0.0331
Glycolic acid	321613.69	199950.60	-1.61	0.0263
Malic acid	291734.82	119909.72	-2.43	0.0114
Cholesterol	56759.19	102280.10	1.80	0.0267
Mannose-6-phosphate	519338.44	106003.63	-4.90	0.0434
Arachidic acid	260462.63	111128.03	-2.34	0.0173
L-Glutamic acid	478500.28	168870.35	-2.83	0.0142
1-Monopalmiti	11585.59	39277.09	3.39	0.0187
inosine	116336.70	23775.23	-4.89	0.0311
L-Lysine	2339317.60	1563038.96	-1.50	0.0419
Pyroglutamic acid	15110123.85	7976423.88	-1.89	0.0277
Stearic acid	363923.99	786258.86	2.16	0.0025
Valeric acid, 5-amino-	630675.10	306205.50	-2.06	0.0413
Phosphoric acid	19315799.99	15441629.09	-1.25	0.0031
α-Cortolone	490875.08	290194.65	-1.69	0.0245
Glyceric acid	65657.38	34671.71	-1.89	0.0021
Uracil	3646033.11	751739.04	-4.85	0.0427
Thymine	194232.78	137217.78	-1.42	0.0245
Serine	108239.27	78871.60	-1.37	0.0464
Carbodiimide	464003.29	591278.67	1.27	0.0454
Campesterol	20150.88	34516.50	1.71	0.0438
Stigmastanol	33034.50	64170.00	1.94	0.0249
Borane	718135.59	1016476.69	1.42	0.0280
beta-Sitosterol	45547.84	138507.52	3.04	0.0241
